# Sex-Dependent Differences in the Elemental Composition of Internal Organs Determined via Total Reflection X-Ray Fluorescence and Inductively Coupled Plasma Optical Emission Spectroscopy

**DOI:** 10.3390/biomedicines12122774

**Published:** 2024-12-05

**Authors:** Aleksandra Wilk, Zuzanna Setkowicz, Katarzyna Matusiak, Eva Margui Grabulosa, Marzena Rugiel, Paula Kasprzyk, Agnieszka Drozdz, Joanna Chwiej

**Affiliations:** 1 Faculty of Physics and Applied Computer Science, AGH University of Krakow, Al. Mickiewicza 30, 30-059 Krakow, Poland; aleksandra.wilk@fis.agh.edu.pl (A.W.); katarzyna.matusiak@fis.agh.edu.pl (K.M.); agnieszka.drozdz@fis.agh.edu.pl (A.D.); 2 Institute of Zoology and Biomedical Research, Jagiellonian University, Ul. Gronostajowa 9, 30-387 Krakow, Poland; 3 Department of Chemistry, University of Girona, C/ M. Aurèlia Capmany 69, 17003 Girona, Spain

**Keywords:** elemental analysis, total reflection X-ray fluorescence (TXRF), inductively coupled plasma optical emission spectroscopy (ICP-OES), internal organs, male and female rats

## Abstract

Background: Research on elemental changes in tissues and organs provides valuable information enabling better understanding of the physiological processes occurring in a living organism, as well as the pathogenesis and course of various diseases. They may also contribute to the development of new, more effective, and safer therapeutic strategies. So far, they have been carried out mainly on male individuals because of the easier planning and conducting of experiments as well as the lower variability of the results in comparison with studies involving females. Methods: The significance of incorporating both sexes in research concerning elemental alterations of tissues may be unveiled by data concerning the influence of sex on the physiological levels of selected elements in various rat organs. Therefore, here we determined and compared the levels of P, S, K, Ca, Fe, Cu, Zn, and Se in brains, hearts, kidneys, livers, and spleens taken from male and female rats. To measure the concentrations of the elements in digested tissue samples, ICP-OES and TXRF methods were utilized. Results: Significant differences between male and female rats were found for all the organs examined, and the concentrations of most of the tested elements were higher in males than females. The exception was Fe, the level of which in the kidneys and liver was higher in female rats. Sex influenced the elemental composition of spleen the most. For the brain, heart, kidneys, and liver, differences were sparse and were found mainly for the heavier elements.

## 1. Introduction

For many years, animal models have played an instrumental role in research on the pathogenesis, progression, and therapies of various diseases. However, relatively recently, attention has been focused on the necessity of conducting such studies with both male and female animals [1,2,3,4]. The identification of differences between sexes, the impact of sex on metabolism, and the potential tailoring of therapies according to those differences seem to be a crucial step in the development of personalized and precision medicine [5,6,7,8,9,10,11]. It has been shown over the years that the differences between males and females do not concern only the reproductive system. Numerous studies point to their existence also in the case of other systems, including pulmonary (i.e., males have larger absolute lung volumes than females) [12,13,14], cardiovascular (i.e., women have smaller left ventricular chambers and, accordingly, lower stroke volumes) [15,16,17], digestive (i.e., women’s saliva differs in the content of bicarbonate and sodium, while men have fewer defense mechanisms that prevent against esophageal reflux disease) [18,19,20,21], lymphatic (i.e., women have more cardiac lymphatic vessels than males) [22,23], and immune system (i.e., females tend to show greater antibody responses, higher B cell numbers, and higher basal immunoglobulin levels than males) [24,25,26]. Significant sex differences are observed also for the neural [27,28,29] and musculoskeletal systems [30,31,32].

The data on the elemental composition of cells, tissues, and organs may be the source of important information about the progression of both physiological and pathological processes occurring in an organism [33,34,35,36,37,38,39,40,41]. The existing literature shows that the elemental balance of tissues may depend on sex. The research on this matter that has been carried out till now, however, has mainly concerned several types of fish, and knowledge on the influence of sex on the elemental composition of mammalian tissues is still very poor [42,43,44,45,46,47]. Therefore, the aim of the present investigation was to identify differences in the elemental composition of the brain, heart, kidneys, liver, and spleen between male and female adult Wistar rats. Two techniques of multi-element analysis were used to achieve the assumed purpose. To determine the content of minor and trace elements in the digested samples of organs, the total reflection X-ray fluorescence (TXRF) method was utilized. In turn, the major elements were determined with inductively coupled plasma optical emission spectroscopy (ICP-OES).

## 2. Materials and Methods

### 2.1. Animals

All methods are reported in this paper in accordance with the ARRIVE guidelines (https://arriveguidelines.org, accessed on 30 August 2024). Two groups of normal Wistar rats were the subject of this study. The first group consisted of 6 male animals, whilst the second consisted of 6 female animals. The culture of the rats as well as all the procedures were carried out in the Laboratory of Experimental Neuropathology at the Institute of Zoology and Biomedical Research of the Jagiellonian University (Krakow, Poland). This was performed in accordance with the international standards and under approval of the 2nd Local Institutional Animal Care and Use Committee in Krakow (agreement no. 318/2020). Throughout their lives, the animals were kept under conditions of controlled temperature (20 ± 2 °C) and lighting (12 h light and dark cycle). They also had unlimited access to food (Labofeed H Standard, Morawski, Poland) and water.

### 2.2. Sample Preparation

On the 60th day of age, the animals were sacrificed by administering Euthasol-Vet (Euthasol-Vet 400 mg/mL, Le Vet B.V., FATRO POLSKA, Poland) at doses appropriate for their weight (as recommended by the manufacturer at 140 mg per kg in a single injection). After perfusion with saline of high analytical purity from each rat, the brain, kidneys, heart, spleen, and liver were taken. The organs were weighed, deeply frozen in liquid nitrogen, and stored in separate sterile Whirl-Pak bags at a temperature not higher than −20 °C.

For elemental analysis, liquid samples are necessary, therefore the tissues were digested. For this purpose, each organ was placed in a separate Teflon vessel (DAP100) together with 5 mL of high-purity 65% nitric acid (Suprapur, Merck Life Science KGaA, Germany) and was subjected to microwave-assisted digestion using the Speed Wave 4 system (Berghof GmbH, Germany) and the settings proposed by the manufacturer for such type of samples.

The certified reference material (CRM) IAEA-A-13, from the International Atomic Energy Agency (IAEA), was employed to study the quality of the results obtained with TXRF and ICP-OES. This material consists of freeze-dried bovine blood sterilized to ensure long-term stability of the material by inhibiting microbial action. Similarly to the tissue samples, the CRM was prepared for measurements by microwave digestion, adding 5 mL of nitric acid to 200 µg of the material.

The quantitative elemental analysis with the TXRF technique was based on the internal standard method. For this purpose, 1000 ppm gallium solution (Gallium ICP standard traceable to SRM from NIST Ga(NO_3_)_3_ in HNO_3_ 2–3%, 1000 mg/L Ga, Certipur^®^, Merck Life Science KGaA, Germany) was added to the entire volume (around 5 mL) of the digested sample and mixed thoroughly. The gallium solution was added in the amount adjusted to every digest individually to achieve a Ga concentration of 100 mg/kg. In the last step, 8 µL of the final solution was deposited on a Super Frost (Menzel Glasser, New Erie Scientific LLC, USA, a subsidiary of Epredia, USA) glass slide.

### 2.3. Apparatus and Measurement Conditions

The concentrations of Fe, Cu, Zn, and Se in the digested organ samples were determined using the TXRF method at the X-ray Fluorescence Laboratory of the Faculty of Physics and Applied Computer Science at the AGH University of Krakow (Poland). For this purpose, a Rigaku Nanohunter II spectrometer (Rigaku Corporation, Japan) equipped with an X-ray tube with a Mo anode was utilized. The tube voltage and current were 50 kV and 12 mA, respectively. The samples deposited on the Super Frost glass slides were measured at a glancing angle of 0.04° for 500 s.

The content of P, S, K, and Ca in the tissue digests was determined with the ICP-OES technique at the Analytical Technical Services Laboratories of the University of Girona (Girona, Spain). For this study, an Agilent 5100 ICP-OES spectrometer (Agilent Technologies, USA) with a radial-torch configuration was used. The RF power was equal to 1200 W, whilst the plasma flow was 12 L/min. The samples were introduced in the plasma gas using a concentric pneumatic nebulizer. The radiation emitted by excited atoms went to the polychromatic wavelength selector and then was registered by the silicon-based multichannel array CCD detector. The wavelength of the analytical lines used to measure P, S, K, and Ca concentrations were 185.827, 182.562, 766.491, and 317.933 nm, respectively.

### 2.4. Quantification and Data Analysis

In the case of the TXRF technique, the content of Fe, Cu, Zn, and Se in the liquid organ samples was determined using the internal standard method. As the internal standard, Ga was chosen, and the concentration *C_x_* of the element *x* was calculated according to Equation (1):

(1) $$C_{x}=\frac{C_{IS}\cdot \;N_{x}}{N_{IS}\cdot \;s_{x}}$$

where

*C_x_*—concentration of the element *x* in the liquid organ sample [μg/g],
*C_IS_*—concentration of the internal standard in the liquid sample [μg/g],
*N_x_*—net pulse number for the element *x* in the sample spectrum [cts],
*N_IS_*—net pulse number for the internal standard in the sample spectrum [cts],
*s_x_*—relative sensitivity for the element *x*.

The limit of the detection *LOD_xj_* of the element *x* in the analyzed organ subsample *j* was calculated via Equation (2):

(2) $${LOD}_{xj}=\frac{3\cdot C_{xj}\cdot \sqrt{N_{BGxj}}}{N_{xj}}$$

where

*C_xj_*—concentration of the element *x* in the organ subsample *j* [μg/g],
*N_BGxj_*—area of the background under the Kα line of the element *x* in the spectrum of subsample *j* [cts],
*N_xj_*—net peak area of the Kα line of the element *x* in the spectrum of subsample *j* [cts].

To estimate the final values of LODs, all the *LOD_xj_* values obtained for particular elements and organs were averaged.

As mentioned, to determine the concentrations of P, S, K, and Ca in the organ samples, the ICP-OES method was utilized. For the needs of the quantitative elemental analysis, matrix-matched standards containing known concentrations of the four examined elements were prepared and measured. Based on the intensities of the chosen emission lines, the calibration curves were prepared and used to determine the unknown concentrations of P, S, K, and Ca in the measured samples.

In the case of the ICP-OES technique, another approach was used to estimate the detection limits of the elements. The *LOD* for each element *y* was calculated via Equation (3):

(3) $${LOD}_{y}=3\cdot {BEC}_{y}\cdot {RSD}_{0y}$$

where

*BEC_y_*—background equivalent concentration of the element *x* [μg/g],
*RSD*_0*y*_—relative standard deviation for the blank of the element *x* [a.u.].

Intra-day and inter-day precisions of the performed measurements were evaluated by performing a series of measurements of reference material IAEA-A-13 (freeze-dried animal blood) within one day and three selected days within one week (three measurements per day), respectively. Both the mentioned validation parameters were calculated as a coefficient of variation.

For the evaluation of the statistical significance of the differences in the elemental composition of the examined organs between rats of different sexes, a non-parametric Mann–Whitney *U* test was applied. The assumed significance level was equal to 0.05, and appropriate calculations were performed with Statistica software version 7.1 (StatSoft Inc., USA).

## 3. Results

### 3.1. Validation of Experimental Methods

The utility of a given experimental method for the quantitative elemental analysis of samples of a particular type can be confirmed via the determination of technique validation parameters such as trueness, precision(s), or detection limits of elements. This was also performed during this study and, based on the measurements of standard reference material or chosen samples, a set of validation parameters was estimated for the ICP-OES and TXRF methods. The obtained data are stated in Table 1, Table 2, Table 3 and Table 4.

A comparison of the element concentrations measured for the reference material IAEA-A-13 with the certified values is presented in Table 1. As one can see, better results of trueness were obtained for the ICP-OES than the TXRF method. The concentrations of Fe, Cu, Zn, and Se determined with the TXRF method demonstrated slightly increased levels in comparison to the range of concentrations declared by the reference material manufacturer. On the other hand, the values of precisions (for details, see Table 2) were satisfactory for both the instrumental methods, and for all of the analyzed elements did not exceed 5%. In the context of reliable detectability and quantification of elements, it is also necessary to mention that the detection limits estimated for most of the elements were several orders of magnitude lower than their content in the tissues (Table 3 and Table 4). The only exception was Se, for which the values of the mentioned validation parameter were around two orders of magnitude lower than the concentration of the element in the tested organs.

### 3.2. Differences in Organ Element Composition Between Male and Female Rats

**Figure 1:**
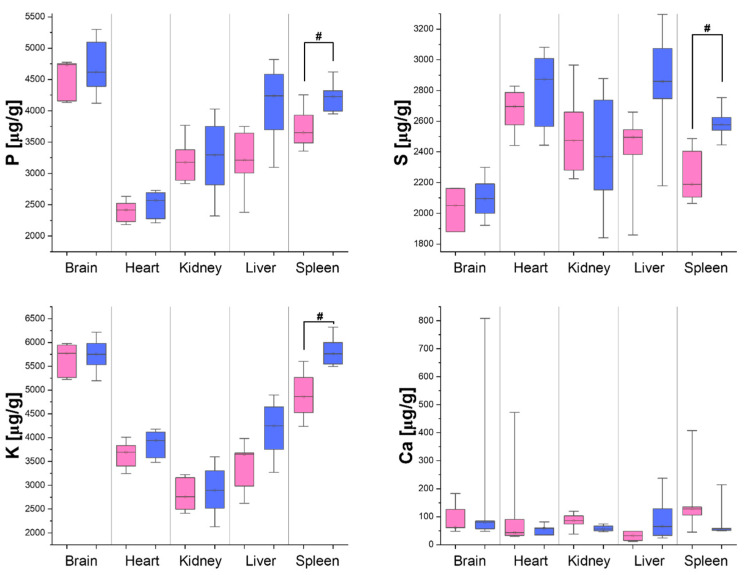
Concentrations of P, S, K, and Ca in the brain, heart, kidneys, liver, and spleen of the male (blue) and female (pink) rats. The median, interquartile range, and minimal and maximal values of the concentrations are indicated. Statistically significant differences in elemental composition between the sexes were identified using the Mann–Whitney *U* test at a significance level of 5%. The concentrations of elements showing significantly higher levels in the male compared to female animals are marked with #.

**Figure 2:**
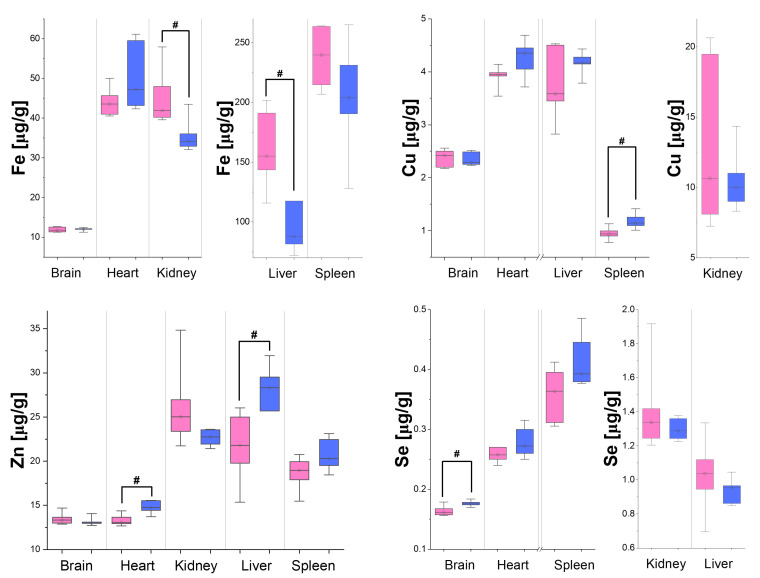
Concentrations of Fe, Cu, Zn, and Se in the brain, heart, kidneys, liver, and spleen of the male (blue) and female (pink) rats. The median, interquartile range, and minimal and maximal values of the concentrations are indicated. Statistically significant differences in elemental composition between the sexes were identified using the Mann–Whitney *U* test at a significance level of 5%. The concentrations of elements showing significant differences between the males and females are marked with #.

The distribution of the concentration of elements in the organs taken from the six male and six female rats is presented in Figure 1 and Figure 2 in the form of box-and-whisker plots showing the medians, maximal and minimal values, as well as interquartile ranges of the concentrations obtained for both sexes. Additionally, in Table 5, the elements presenting concentrations significantly higher in organs taken from males than females are marked with upward arrows. The opposite relations, observed for Fe, are marked with downward arrows.

As one can notice, comparing the results presented in Figure 1 and Figure 2 with the values of the LODs (Table 3 and Table 4), all the measured elements displayed concentrations high enough to enable their quantitative comparisons. Differences in elemental composition between the female and male rats were observed for all the organs examined. The concentrations of most of the tested elements were higher in the male than female rats (see Table 5). The exception was iron, the level of which in the kidneys and liver was higher in the females than in the males.

## 4. Discussion

The main motivation for this study was the fact that knowledge on sex-dependent differences in the accumulation of major, minor, and trace elements in rat tissues is very poor. In Table A1 of Appendix A, we have summarized literature data concerning the physiological levels of the elements, being the subject of this study, in the organs of male and female rats that have been published so far [48,49,50,51,52,53,54,55,56]. In the cited works, the elemental analyses were mostly carried out in terms of the changes characteristic for specific disease and therefore usually concentrated on only one tissue type [53,56]. Additionally, the most common practice was to concern only male specimens in the studies [49,50,51,52]. The only exception to this practice was investigations that deal with sex-specific conditions [57,58].

To the best of our knowledge, there is only one paper considering the influence of sex on the elemental composition of rat tissues. Healthy animals, being the subject of this investigation, were, however, fed with a very specific semisynthetic fodder [59], and therefore, the results of the mentioned study should not be compared with our and other works, where naive normal rats on a standard laboratory diet were used.

Moreover, the analysis of the data presented in Table A1 of Appendix A points not only to a small number of papers dealing with the subject of this study but also great discrepancies in the measured concentrations of some elements. Especially huge discrepancies in the experimental data were found for Ca, whose levels in normal organs/tissues sometimes differed in the order of magnitude or even more [48,53,56].

TXRF spectroscopy, which was used in our study, is an instrumental technique of multi-element analysis. The wide range of examined concentrations, low detection limits, small amount of sample required for the measurement, as well as cost-effectiveness are the main advantages that result in its wide usage in many fields of science [48,60,61,62,63,64]. However, this method also has its limitations, particularly the poorer accuracy and precision in the determination of concentrations for lighter elements [65,66,67,68]. Therefore, in the present study, the TXRF method was used to measure the concentrations of Fe, Cu, Zn, and Se in the digested tissue samples. In turn, the elements with the lower Z, such as P, S, K and Ca, were determined with the ICP-OES method [69,70,71].

P constitutes a building block of nucleic acids, phospholipids, phosphoproteins, and ATP. It is involved in many processes taking place in cells and tissues. The homeostasis of P in the body is regulated hormonally. The parathyroid hormone causes the release of phosphates from the bones and stimulates kidneys to remove them from the body [72,73]. The existing literature evidence points to the significant influence of estrogen on P levels in tissues. Carillo-Lopez et al. and Takasugi et al. showed that estrogens increase the mRNA expression and protein level of fibroblast growth factor FGF23 in osteoblast-like cells [73,74,75]. FGF23 increases the excretion of phosphate through the kidneys and regulates its serum contents. Therefore, the lower P in the spleen and liver (differences not statistically relevant, *p*-value = 0.06) of the female rats observed in this study (relative, compared to males, difference in medians of 14% for the spleen and 24% for the liver) might be connected with the higher estrogen levels in their organisms. Although low level of serum P is often linked with the dysfunction of neural tissue and is mentioned as a risk factor for the development of Alzheimer’s disease, we did not detect any differences in the concentration of this element in the brain between the male and female rats [76,77].

S is one of the most abundant elements in the human body, and its presence is associated with sulfur-containing amino acids: methionine, cysteine, cystine, homocysteine, and taurine. Functionally, S is crucial for the synthesis and recycling of an important antioxidant glutathione. Additionally, this element plays a role in maintaining the integrity of connective tissues like the skin, tendons, and ligaments [78,79]. It was discovered and described by Melnik that sex hormones have a direct impact on serum homocysteine and cysteine levels [79]. It was found that high testosterone and low estradiol levels are associated with elevated homocysteine and cysteine levels as well as diminished hydrogen sulfide levels in serum. The mentioned observations seem to be in line with our results that revealed lower concentrations of S in the spleens and livers (differences not statistically relevant, *p*-value = 0.06) that were taken from the female rats (relative, compared to males, difference in medians of 14.3% for the spleen and 13.0% for the liver) [80,81].

Sex-dependent differences in K accumulation were analogical to those observed for P and S. K, the main intracellular cation in the body, is essential for maintaining a normal cell resting membrane potential and for generating and propagating action potentials in excitable tissues. The hormone responsible for the regulation of potassium levels is aldosterone [82]. Aldosterone acts in the renal tubules, causing the body to retain sodium and water. Simultaneously, it increases the excretion of potassium in the urine, which diminishes the content of the element in the serum. As described by Rossi et al., estrogen receptors play an important role in controlling aldosterone biosynthesis in the glomerular zone of the adrenal cortex, and high estrogen levels in pre-menopausal women may tonically impair this hormone synthesis [83,84,85].

The function of Ca in the body is not limited only to the bone formation; the element also plays an important role in the process of blood coagulation, muscle contraction, the functioning of the nervous system, and the regulation of normal heart rhythms [86,87]. Our study did not detect any statistically significant differences in Ca concentration within the tested organs between the male and female individuals. However, it is worth mentioning that Ca levels may vary substantially in overall tissue volume, due to the tendency of carbonates to form crystals and calcification in the situation that their levels are high [88].

K and Ca play an important role in maintaining cell membrane potential. A deficiency or excess of membrane transmitters is associated with a disruption of membrane signaling and thus cellular dysfunction. The results of our study seem to point to the influence of estrogen on the electrolyte concentrations in the examined organs. Although our study did not detect statistically significant differences in the tissue calcium concentration between rats of different sexes, female organs showed slightly higher calcium levels (relative, compared to males, difference in medians of 53.6% for the kidneys and 32.7% for the spleen). This is consistent with other literature data which indicate that estrogen increases calcium absorption in the human body [89]. In addition, it affects the production of aldosterone and thus potassium metabolism [90].

Fe is present in human body in hemoglobin, myoglobin, and various enzymes. Thus, it participates in the transport of oxygen, its storage in the muscles, as well as in the production of energy and the regulation of various cellular functions, including proliferation [91,92]. Sex-dependent differences in iron levels in the blood and tissues may be due to many factors, but the most important seems to be the dissimilar hepcidin expressions in the liver of males and females. Hepcidin inhibits Fe transport through binding to ferroportin located in the plasma membrane of macrophages and gut enterocytes. Ferroportin inhibition in macrophages causes Fe sequestration within the cell, whilst in enterocytes, it reduces the dietary absorption of Fe [92,93,94]. As shown by Harrison-Findik, female mice show significantly higher hepcidin expression in the liver than males [93]. These elevated hepcidin levels may be just responsible for the higher Fe concentrations observed in the kidneys and liver of female rats (relative, compared to males, difference in medians of 23.2% for the kidneys and 76% for the liver).

In the human body, Cu is essential for the proper functioning of enzymes involved in aerobic metabolism, such as cytochrome c oxidase in the mitochondria. Together with iron, this element is involved in the formation of red blood cells [95,96]. As shown by Quinn et al., female mice and women exhibit higher absorption of copper in the intestine, thus translating into its higher levels in the blood [97,98]. Altered ceruloplasmin and copper concentrations were also observed in patients taking oral contraceptives [98,99]. Assuming that, in non-pregnant female rats, the influence of estrogen on Cu levels is negligible, higher levels of the element in the spleen of male rats (relative, compared to males, difference in medians of 18.3%) may rather be an effect of increased testosterone levels.

The role of zinc in the body is closely related to those of iron and copper. Zinc is essential for immune cell development and function, hematopoiesis, cell signaling, and inflammatory response. It supports the proper functioning of the ovaries and is essential for sperm production and maintaining healthy testosterone levels [100,101]. It has been shown by Rea that sex has influence on the level of Zn in blood overall. Male patients presented higher blood Zn levels, which may translate to higher tissue Zn levels, in comparison to females [102]. Although the mentioned effect is not well understood yet, it may be responsible for the lower concentration of Zn noticed by us in the organs of the female rats (relative, compared to males, difference in medians of 11.3% for the heart, 23% for the liver, and 6.6% for the spleen).

Se in human body plays roles analogical to those of Cu and Zn; it protects against oxidative stress, endoplasmic reticulum stress, and inflammation [103,104]. It is also essential for sperm production and maintaining healthy testosterone levels. Seale et al. have highlighted the importance of sex on selenium metabolism and demand for this element. It was shown that high Se levels are tightly bound to adequate testosterone production and male development from the first days of life [103]. Assuming healthy animals were taken into consideration in this research, the slightly higher Se concentration in male brain tissue can be explained by this phenomenon (relative, compared to males, difference in medians of 8.0%) [103].

Both Se and Zn are important antioxidants in the body. The regulation of their absorption and release in tissues is closely related to the level of testosterone present in the body. The higher concentrations of these elements recorded in our study in males seem to be in agreement with the literature, as high testosterone levels are associated with higher concentrations of Zn and Se in the blood. On the other hand, it is also known that supplementation of these elements positively affects testosterone levels in the body and fertility of males [102,105,106].

## 5. Conclusions

The aim of this work was to determine the differences in the elemental composition of selected organs between male and female rats. Knowledge in this topic is still very poor. Most of the investigations to date have focused on one specific tissue, usually in the context of one particular disease, or on the metabolism of one selected element. Additionally, when sex is not a critical factor for the examined medical problem, the studies are mostly performed on male specimens. Our results clearly point to differences in the elemental composition of the internal organs between the normal male and female rats as well as at the natural population variability occurring in healthy specimens. At the same time, they confirm the huge importance of the incorporation of both sexes of experimental animals in in vivo research. This seems to be especially important when studies concern elemental changes, based on which the conclusions on physiological or pathological processes occurring in the living organism are based. It should be emphasized that our study was carried out on a limited number of animals; thus, further investigations on larger populations as well as more detailed analyses of element distributions in the examined organs are necessary.

## Figures and Tables

**Table 1:** A comparison of the element concentrations obtained with the ICP-OES (P, S, K, and Ca) and TXRF (Fe, Cu, Zn, and Se) methods for the reference material IAEA-A-13 with the certified values.

Element	Certified Value [µg/g] *	Measured Value [µg/g]
P	690–1120	778
S	6000–7000	6384
K	2100–2700	2790
Ca	226–332	322
Fe	2200–2500	3147
Cu	3.7–4.8	5.17
Zn	12–14	26.16
Se	0.15–0.31	0.48

* 95% confidence interval.

**Table 2:** The intra- and inter-day precision values obtained for ICP-OES (P, S, K, and Ca) and TXRF (Fe, Cu, Zn, and Se) methods based on the measurements of the reference material IAEA-A-13.

Element	Intra-Day Precision [%] *	Inter-Day Precision [%]
P	1.19	0.89
S	0.96	1.08
K	1.04	2.38
Ca	2.13	0.98
Fe	0.42	0.35
Cu	1.58	1.37
Zn	3.36	1.65
Se	4.44	2.91

* The intra- and inter-day precision values were calculated as coefficients of variation.

**Table 3:** The detection limits of the elements in [μg/g] estimated for particular organs with the TXRF method.

Element	Brain	Heart	Kidneys	Liver	Spleen
Fe	0.0551 (0.0020)	0.0647 (0.0036)	0.0695 (0.0036)	0.0756 (0.0031)	0.1351 (0.0057)
Cu	0.0335 (0.0014)	0.0380 (0.0021)	0.0450 (0.0027)	0.0369 (0.0015)	0.0562 (0.0024)
Zn	0.0425 (0.0017)	0.0513 (0.0030)	0.0640 (0.0037)	0.0502 (0.0020)	0.0697 (0.0028)
Se	0.01848 (0.00072)	0.0195 (0.0011)	0.0226 (0.0014)	0.01936 (0.00077)	0.0304 (0.0012)

**Table 4:** The detection limits of the elements in [μg/g] estimated for the ICP-OES method.

Element	LOD (SD) [μg/g]
P	0.0217 (0.0045)
S	0.059 (0.015)
K	0.0242 (0.0087)
Ca	0.0144 (0.0038)

**Table 5:** Statistically relevant differences in the elemental composition of the examined organs between the male and female individuals.

Tissue/Element	P	S	K	Ca	Fe	Cu	Zn	Se
Brain	-	-	-	-	-	-	-	↑
Heart	-	-	-	-	-	-	↑	-
Kidneys	-	-	-	-	↓	-	-	-
Liver	-	-	-	-	↓	-	↑	-
Spleen	↑	↑	↑	-	-	↑	-	-

## Data Availability

The data supporting the conclusions of this article will be made available by the corresponding author on reasonable request.

## Appendix A

**Table A1:** Literature data on concentrations of P, S, K, Ca, Fe, Cu, Zn, and Se in chosen rat organs for male and female rats [48,49,50,51,52,53,54,55,56].

Element/Tissue	Concentration of Element in Tissue [µg/g]											
	Brain		Heart		Kidneys		Liver		Spleen		Literature	
	M	F	M	F	M	F	M	F	M	F	M	F
P	2519–6110	-	2035–2970	-	2529–4250	3280–6034	2981–5750	3380–6542	3185–5460	6997–7439	[48,49]	[53,54,55]
S	1312–2030	-	2490–2529	-	2290–2331	3924–4386	2351–2980	2390–4192	1957–2490	3705–4476	[48,49]	[53,54,55]
K	3092–6100	-	2768–3470	-	2473–2920	2580–5668	3205–5440	3000–7294	4045–5290	7865–9098	[48,49,50]	[53,55]
Ca	33–730	-	26–112	-	55–120	72–291	30–1300	32.3–159.0	33–2070	124–236	[48,49,50]	[53,56]
Fe	11.4–20.6	-	57–75	445–674	40–45	103–513	71–137	278–1023	185–547	592–818	[48,49,51]	[53,54,55,56]
Cu	1.94–3.78	-	4.49–9.04	18.4–21.6	6.37–8.84	16.0–48.7	3.16–8.37	5.4–17.0	1.10–2.34	3.03–4.09	[48,49,52]	[53,54,55,56]
Zn	10.8–50.0	-	14.6–25.4	62.6–69.1	18.9–32.0	29.0–80.4	29.4–125.0	32.3–103.0	20.4–159.0	30–57	[48,49,52]	[53,54,55,56]
Se	0.127–0.193	-	0.364–0.430	-	1.40–1.90	2.5–3.1	0.59–1.60	2.32–3.23	0.42–0.54	0.64–0.81	[48,50,51]	[53,54,55,56]
